# Immune Thrombocytopenic Purpura as Initial Presentation of Paediatric SLE: A Case Report

**DOI:** 10.31729/jnma.5591

**Published:** 2021-01-31

**Authors:** Anjila Ghimire

**Affiliations:** 1 Department of Clinical Oncology, Bir Hospital, Kathmandu, Nepal

**Keywords:** *female child*, *immune thrombocytopenia*, *pediatric SLE*

## Abstract

The systemic lupus erythematosus (SLE) is a connective tissue disorder with variable presentations in children. The usual presentation includes arthritis, malar rash, nephritis, hemolytic anemia, and fever. Isolated hematologic abnormality as the only presentation of SLE is rare. Here is a case report of a female child presented to us with superficial and mucosal bleeding with isolated low platelet count and anemia in proportion to blood loss. When platelet count didn't go up despite appropriate treatment in lines of ITP, further investigations were done, diagnosis of SLE was established, and management was done accordingly.

## INTRODUCTION

The isolated hematological manifestation of SLE in children is a rare entity, and it occurs in the form of hemolytic anemia, thrombocytopenia, and persistent leukopenia.^1^ The median age at diagnosis is 11-12 years, and about 20% of all individuals with SLE are diagnosed before 16 years of age.^2^ Here is a case report of an eight-year female child who had isolated thrombocytopenia and bleeding manifestations and did not respond to standard treatment of Immune thrombocytopenic purpura. Further evaluation revealed the diagnosis of SLE. A high index of suspicion is thus necessary while managing such treatment-refractory cases of ITP to reach the correct diagnosis.^3^

## CASE REPORT

Eight year female presented to the Department of Paediatrics, BPKIHS with the complaint of painless pinpoint bleeding spots and bluish patches over different parts of the body for 1 month. Lesions were reddish in color distributed over the face, oral cavity, upper and lower limbs, and torso. She also had reddish discoloration of the sclera. The lesions were self-limiting in nature and disappeared over a period of about three weeks initially only to recur a week later with bleeding from gums, blood in urine, and stool. When the gum bleeding didn't subside, she was brought to medical attention. Throughout this time, there was no fever, weight loss, swelling of gums, excessive sweating, bone pain, joint pain, joint swelling, or deformity.

On examination, the child was pale. Petechiae, purpura, and ecchymosis were distributed all over the body. Purpura over the palate and subconjunctival hemorrhage was seen. There was no lymphadenopathy, joint swelling, or organomegaly.

Initial Investigations revealed anemia with thrombocytopenia. The total white cell count was normal. Peripheral blood smear revealed normocytic normochromic anemia with thrombocytopenia. Bone marrow biopsy revealed megakaryocytic thrombocytopenia. Blood culture showed no growth. Liver function test, renal function test, LDH, coagulation parameters were within normal limits. Serology for HIV I/II, Hepatitis B, and C was non-reactive.

The child was treated with anti D immunoglobulin in lines of acute ITP. There was a rise in platelet count up to 36,000/μl. There were no new lesions, and previous lesions had resolved. Anemia was treated. The patient was discharged on day 4 with oral prednisolone at 1mg/kg/day, and follow-up was scheduled after three days.

There was a drop in platelet count to 7000/μl. The child was readmitted and given IVIG at 0.8g/kg. There was a rise in platelet count up to 96000/μl, but it fell and remained at 10,000/μl. This time, she was further evaluated in SLE lines regarding thrombocytopenia as the hematologic manifestation of the disease. ANA, dsDNA was elevated with the decrease in C3 and C4 levels.

**Table 1:** Blood counts at the time of presentation

Parameter	value	Reference range
Haemoglobin	7.0g/dl	11-13g/dl
Total WBC count	7600	4000-11000/μl
Platelet count	5000/μl	150000-400000/μl
Peripheral blood smear	Normocytic, normochromic anaemia with platelet count <10000	

**Table 2:** Investigations done in the second setting.

parameters	Result	Lab reference
C3	52 mg/dl (cobas)	90-180mg/dl
C4	4mg/dl (cobas)	10-40mg/dl
ANA	228AU/ml	>10 AU/ml
ds-DNA	38.56 IU/ml	>10IU/ml

She was kept on IV Methylprednisolone at 30mg/kg for three days followed by oral prednisolone at 2mg/ kg/day to continue until regaining platelet count to >100000/μl. Platelet count increased to 102,000/μl on day 8, and she was discharged. Thus, a diagnosis of juvenile SLE with hematologic manifestations without other organ system involvement was made.

The plan was to taper steroids slowly and evaluate for the introduction of steroid-sparing drugs at follow up.

## DISCUSSION

SLE in the pediatric population usually presents fever, fatigue, skin involvement, hematologic abnormalities, and mucosal ulceration, but atypical presentations can occur. In our scenario, the patient had only thrombocytopenia, and anemia was consistent with blood loss. A case report by A Tamaddoni et al. in 2015 describes an adolescent boy with ITP as the presenting feature of SLE. There was associated reactive lymphadenopathy.^4^ A case of pediatric lupus with neuropsychiatric manifestations in addition to thrombocytopenia was reported by Sankar Raj in 2013.^5^

Studies have been done in the adult population where ITP was the initial presentation of SLE. A study by Peralta M et al. in 1997 concluded that 12.1% of cases of acute ITP developed SLE during a follow-up of 7.2 years.^6^ Study done by So Eun Jun et al. in 2008 in Korea showed that ANA positivity in ITP could be as high as 20.8% through progression to SLE was infrequent.^7^ Risk factors for the future development of SLE in ITP patients were studied by Hazzan et al. in 2006. Female sex, age group 12.7±3.6 years, high ANA titers, and chronic ITP were the ones who developed SLE during the follow-up period of 4.2±4.9 years.^8^

**Figure:**
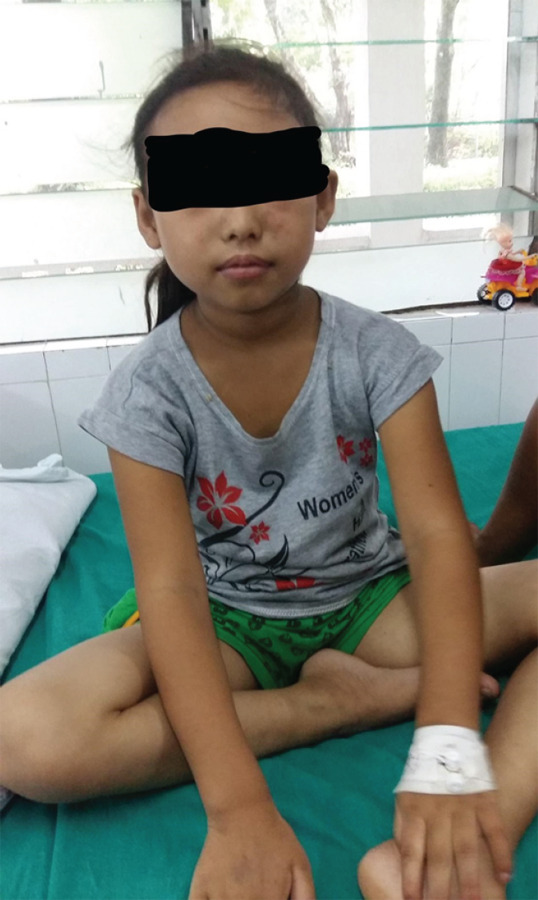


Paediatric SLE could present with atypical features, so a high index of suspicion is needed for diagnosis, especially in adolescent females. It is essential to diagnose and initiate appropriate treatment in time to avoid adverse effects of the disease on growth and halt the progression of the disease. Our report showed that isolated thrombocytopenia refractory to treatment with IVIG, anti D, and a conventional dose of steroids could be the initial SLE presentation; thus, timely assessing ANA and ds-DNA levels seems crucial for recognizing SLE as the primary disease. Also, ANA positivity could be there in a cluster of patients of ITP. They need to be followed up for progression to SLE.
